# Quantum Blobs

**DOI:** 10.1007/s10701-012-9636-x

**Published:** 2012-02-29

**Authors:** Maurice A. de Gosson

**Affiliations:** Faculty of Mathematics, University of Vienna, NuHAG, 1090 Vienna, Austria

**Keywords:** Quantum phase space, Symplectic capacity, Coherent states, Wigner function, Fermi’s function

## Abstract

Quantum blobs are the smallest phase space units of phase space compatible with the uncertainty principle of quantum mechanics and having the symplectic group as group of symmetries. Quantum blobs are in a bijective correspondence with the squeezed coherent states from standard quantum mechanics, of which they are a phase space picture. This allows us to propose a substitute for phase space in quantum mechanics. We study the relationship between quantum blobs with a certain class of level sets defined by Fermi for the purpose of representing geometrically quantum states.

## Introduction

### Basil Hiley and Me

It is indeed an honour and a pleasure to Contribute to Basil Hiley’s Festschrift. When I met Basil for the first time (it was in the late 90s, during my Swedish exile) I was immediately fascinated not only by his vision of quantum mechanics and its philosophy, but also by the man himself; I was immediately charmed by his utterly unassuming and gentlemanly manners together with his British humour. Basil patiently explained to me the subtleties of the causal interpretation of quantum mechanics and of the Implicate Order; our conversations were invariably accompanied by a cup of strong Assam tea, his favourite beverage (during daytime, that is; later at night we occasionally replaced the cup of tea by a glass of a beverage known in France as *Pastis*). Of course, I already had read a lot about the causal interpretation of quantum mechanics, but my knowledge and understanding of this theory was merely on an abstract mathematical level. Thanks to Basil’s pedagogical skills *Physics* now entered the scene and helped me to understand some of the deep implications of the causal interpretation. However, Basil also was a patient and empathetic listener, always eager to hear about new developments in mathematics (Basil is not only a brilliant physicist, he also has an excellent taste for mathematics). When I explained to him my ideas on the uncertainty principle and introduced him to the “symplectic camel” and “quantum blobs”, he immediately became very enthusiastic and encouraged me to pursue the approach I had initiated in some recent papers. He was even kind enough to honour me by writing a foreword to my book [6] where I explained some of these ideas. Therefore I could do no less than to write this modest contribution to the “Hiley Festschrift” as a tribute to my friend Basil for his Helsinki birthday party!

### Contents

In this paper I establish a fundamental correspondence of a geometric nature between the squeezed coherent states familiar from quantum optics, and quantum blobs. The latter are related the principle of the symplectic camel, which is a deep topological property of canonical transformations, and allow a “coarse graining” of phase space in units which are symplectic deformations of phase space balls with radius $\sqrt{\hbar}$.

This paper is structured as follows. I begin by reviewing in Sect. 1 the main definitions and properties of squeezed coherent states; in Sect. 2 I introduce the notion of quantum blob which we discuss from a purely geometric point of view. In Sect. 3 the fundamental correspondence between squeezed coherent states and quantum blobs is established; this correspondence which is denoted by $\mathcal{G}$ is bijective (that is one-to-one and onto); its definition is made possible using the theory of the Wigner transform of Gaussian functions. In Sect. 4 I prove the fundamental statistical property of quantum blobs: they are a geometric picture of minimum uncertainty. Finally, in Sect. 5, I shortly discuss the relationship between quantum blobs and a certain level set introduced in 1930 by Enrico Fermi and which seems to have been almost unnoticed in the Scientific literature. The paper ends with some conjectures and a discussion of related topics I plan to develop in further work.

### Notation

The phase space $\mathbb{R}_{z}^{2n}\equiv\mathbb{R}_{x}^{n}\oplus \mathbb{R}_{p}^{n}$ (*n*≥1) is equipped with the standard symplectic form *σ*(*z*,*z*′)=*p*⋅*x*′−*p*′⋅*x* if *z*=(*x*,*p*), *z*′=(*x*′,*p*′). We are writing *x*=(*x*_1_,…,*x*_*n*_), *p*=(*p*_1_,…,*p*_*n*_), and *p*⋅*x*=*p*_1_ *x*_1_+⋯+*p*_*n*_ *x*_*n*_ is the usual Euclidean scalar product of *p* and *x*. Equivalently *σ*(*z*,*z*′)=*Jz*⋅*z*′ where  is the standard symplectic matrix. The group of linear automorphisms of $\mathbb{R}_{z}^{2n}$ is denoted by $\operatorname{Sp}(2n,\mathbb{R})$ and called the standard symplectic group. We have $S\in\operatorname{Sp}(2n,\mathbb{R})$ if and only if *S* is a linear mapping $\mathbb{R}_{z}^{2n}\longrightarrow\mathbb{R}_{z}^{2n}$ such that *S* ^*T*^ *JS*=*J*.

## Squeezed Coherent States

For details and complements see the seminal paper by Littlejohn [26]; Folland [18] also contains valuable information.

The archetypical example is that of the fiducial (or standard, or vacuum) coherent state

1 $$\Phi^{\hbar}(x)=(\pi\hbar)^{-n/4}e^{-|x|^{2}/2\hbar}, $$

where the factor (*πħ*)^−*n*/4^ is introduced in order to ensure normalization. It was first systematically used by Schrödinger in 1926. The notation Φ^*ħ*^(*x*)=〈*x*|0〉 is also widely used in quantum mechanics. It represents the ground state of the isotropic harmonic oscillator; alternatively it is an eigenstate of the annihilation operator with eigenvalue zero. More generally one wants to consider Gaussians of the type

2 $$\Phi_{X,Y}^{\hbar}(x)=(\pi\hbar)^{-n/4}(\det X)^{1/4}e^{-\frac{1}{2\hbar }(X+iY)x\cdot x}, $$

where *X* and *Y* are real symmetric *n*×*n* matrices, *X* positive definite; we have $\|\Phi_{X,Y}^{\hbar}\|_{L^{2}}=1$. The Gaussian (2) is called a squeezed (or generalized) coherent state. We can still go one step further and define the shifted squeezed coherent state

3 $$\Phi_{X,Y,z_{0}}^{\hbar}(x)=\widehat{T}^{\hbar}(z_{0})\Phi_{X,Y}^{\hbar}(x), $$

where $\widehat{T}^{\hbar}(z_{0})$ is the Heisenberg–Weyl operator: if Ψ is a function on configuration space $\mathbb{R}_{x}^{n}$ then

4 $$\widehat{T}^{\hbar}(z_{0})\Psi(x)=e^{\frac{i}{\hbar}(p_{0}\cdot x-\frac{1}{2}p_{0}\cdot x_{0})}\Psi(x-x_{0}). $$

We will write from now on

5 $$M=X+iY, \quad \Phi_{M}^{\hbar}=\Phi_{X,Y}^{\hbar},\quad \Phi_{M,z_{0}}^{\hbar}=\widehat{T}^{\hbar}(z_{0})\Phi_{M}^{\hbar}. $$

The important thing is that squeezed coherent states are naturally obtained from the fiducial state (1) by letting metaplectic operators act on it. Let us explain this property shortly; for details see for instance de Gosson [11]. The symplectic group $\operatorname{Sp}(2n,\mathbb{R})$ has a covering group of order two, the metaplectic group $\operatorname{Mp}(2n,\mathbb{R})$. That group consists of unitary operators (the metaplectic operators) acting on *L* ^2^(ℝ^*n*^). There are several equivalent ways to describe the metaplectic operators. For our purposes the most tractable is the following: assume that $S\in\operatorname{Sp}(2n,\mathbb{R})$ has the block-matrix form

6 $$S=\begin{pmatrix}A & B\\C & D\end{pmatrix} \quad \text{with\ }\det B\neq0. $$

The condition det*B*≠0 is not very restrictive, because one shows (de Gosson [6, 11]) that every $S\in\operatorname{Sp}(2n,\mathbb{R})$ can be written (non uniquely however) as the product of two symplectic matrices of the type above; moreover the symplectic matrices arising as Jacobian matrices of Hamiltonian flows determined by physical Hamiltonians of the type “kinetic energy plus potential” are of this type for almost every time *t*. To the matrix (6) we associate the following quantities:

- A quadratic form
  $$W\bigl(x,x^{\prime}\bigr)=\frac{1}{2}DB^{-1}x\cdot x-B^{-1}x\cdot x^{\prime}+\frac{1}{2}B^{-1}Ax^{\prime}\cdot x^{\prime}$$
  defined on the double configuration space $\mathbb{R}_{x}^{n}\times \mathbb{R}_{x}^{n}$; the matrices *DB* ^−1^ and *B* ^−1^ *A* are symmetric because *S* is symplectic (*W*(*x*,*x*′) is often called “Hamilton’s characteristic function” Goldstein [19]) in mechanics, or “eikonal” in optics; it is closely related to the notion of action de Gosson [6, 11]);
- The complex number $\Delta(W)=i^{m}\sqrt{|\det B^{-1}|}$ where *m* (“Maslov index”) is chosen in the following way: *m*=0 or 2 if det*B* ^−1^>0 and *m*=1 or 3 if det*B* ^−1^<0.

The two metaplectic operators associated to *S* are then given by

7 $$\widehat{S}\Psi(x)= \biggl( \frac{1}{2\pi i} \biggr)^{n/2}\Delta(W)\int e^{\frac{i}{\hbar}W(x,x^{\prime})}\Psi\bigl(x^{\prime}\bigr)d^{n}x^{\prime}. $$

The fact that we have two possible choices for the Maslov index shows that the metaplectic operators occur in pairs $\pm\widehat{S}$; this of course is just a reflection of the fact that $\operatorname{Mp}(2n,\mathbb{R})$ is a two-fold covering group of $\operatorname{Sp}(2n,\mathbb{R})$.

The action of $\operatorname{Mp}(2n,\mathbb{R})$ on squeezed coherent states is given by the following result:

### Proposition 1

*Let* $\widehat{S}\in\operatorname{Mp}(2n,\mathbb{R})$ *be one of the two metaplectic operators corresponding to the symplectic matrix*  (*we do not make the assumption* det*B*≠0). *Then*

8 $$\widehat{S}\Phi_{M}^{\hbar}=e^{\frac{i}{\hbar}\gamma(\widehat{S})}\Phi_{M_{S}}^{\hbar} \quad \text{\textit{with}\ }M_{S}=i(AM+iB)(CM+iD)^{-1} $$

*where* $e^{\frac{i}{\hbar}\gamma(\widehat{S})}$ *is a phase factor such that* $\gamma(-\widehat{S})=\gamma(\widehat{S})+i\pi\hbar$ [*the matrix* *CM*+*iD* *is never singular*]. *More generally we have*:

9 $$\widehat{S}\Phi_{M,z_{0}}^{\hbar}=e^{\frac{i}{\hbar}\gamma(\widehat{S})}\Phi_{M_{S},Sz_{0}}^{\hbar}. $$

### Proof

See Folland [18], Littlejohn [26]. □

This important result motivates the following definition:

### Definition 2

The set $\mathcal{CS}(n,\mathbb{R})$ of all squeezed coherent states consists of all $\{e^{\frac{i}{\hbar}\gamma}\Phi_{M,z_{0}}^{\hbar}\}$ where *γ* is an arbitrary real phase.

We thus do not distinguish between $e^{\frac{i}{\hbar}\gamma}\Phi_{M,z_{0}}^{\hbar}$ and $e^{\frac{i}{\hbar}\gamma^{\prime}}\Phi_{M,z_{0}}^{\hbar}$; we will often omit the prefactor $e^{\frac{i}{\hbar}\gamma}$. Proposition 1 can now be restated in terms of a group action: 


We will come back to this action in a moment and give a geometric picture of it in terms of phase space ellipsoids.

An important property of Proposition 1 above is that $\mathcal{CS}(n,\mathbb{R})$ is preserved by Hamiltonian flows arising from quadratic Hamiltonian functions, i.e. Hamiltonians of the general type

10 $$H(z)=\frac{1}{2}Rz\cdot z, $$

where *R* is a real symmetric matrix. When *H* is of the physical type “kinetic energy plus potential” this amounts considering potentials which are quadratic forms $\frac{1}{2}\Omega x\cdot x$ in the position variables (generalized harmonic oscillator):

11 $$H(z)=\frac{1}{2m}|p|^{2}+\frac{1}{2}\Omega x\cdot x. $$

For Hamiltonians of the type (10) the flow determined by the Hamilton equations

12 $$\dot{x}=\nabla_{p}H(x,p), \quad \dot{p}=-\nabla_{x}H(x,p) $$

consists of linear canonical transformations (Arnol’d [1], Goldstein [19], de Gosson [11]). In fact, rewriting these equations in the form $\dot{z}=JXz$ with *X*=−*JR* the explicit solution is given by *z*_*t*_=(*x*_*t*_,*p*_*t*_)=*e* ^*tX*^ *z*_0_. The matrix *X* belongs to the symplectic Lie algebra $\mathfrak{sp}(2n,\mathbb{R})$ (because *XJ*+*JX* ^*T*^=0, see Folland [18] or de Gosson [6, 11]) hence the matrices *S*_*t*_=*e* ^*tX*^ are symplectic. For instance, for the generalized harmonic oscillator (11) the Hamilton equations are $\dot{x}=p/m$ and $\dot {p}=-\Omega x$ and we have .

It follows from the theory of the metaplectic group that together with the theory of covering spaces (see e.g. Folland [18], de Gosson [11]) that to the path *t*↦*S*_*t*_=*e* ^*tX*^ of symplectic matrices corresponds a unique path $t\longmapsto\widehat{S}_{t}$ of metaplectic operators such that $\widehat{S}_{0}$ is the identity. The remarkable fact is that this family of operators $\widehat{S}_{t}$ is just precisely the quantum flow determined by Schrödinger’s equation

13 $$i\hbar\frac{\partial\Psi}{\partial t}=H(x,-i\hbar\nabla_{x})\Psi, $$

where *H*(*x*,−*iħ*∇_*x*_) is the (Weyl) quantization of the quadratic Hamiltonian (10); for instance when *H* has the physical type (11) this equation is just the usual equation

14 $$i\hbar\frac{\partial\Psi}{\partial t}= \biggl[ -\frac{\hbar^{2}}{2m}\nabla_{x}^{2}+\frac{1}{2}\Omega x\cdot x \biggr] \Psi. $$

Thus the solution of (13) is given by the simple formula

15 $$\Psi(x,t)=\widehat{S}_{t}\Psi_{0}(x) ,\quad \Psi_{0}(x)=\Psi(x,0). $$

In particular, if the initial wavefunction Ψ_0_(*x*) is a coherent state $\Phi_{M_{0},z_{0}}^{\hbar}$ Proposition 1 shows that the solution Ψ(*x*,*t*) is explicitly given by

16 $$\Psi(x,t)=e^{\frac{i}{\hbar}\mathcal{\gamma}(t)}\Phi_{M_{t},z_{t}}^{\hbar}(x) $$

- *z*_*t*_=(*x*_*t*_,*p*_*t*_) is the solution of Hamilton’s equations $\dot {x}=\nabla_{x}H$, $\dot{p}=-\nabla_{p}H$ passing through the point *z*_0_ at time *t*=0;
- *M*_*t*_ is calculated using formula (8): write *S*_*t*_ as a symplectic block matrix ; then
  17 $$M_{t}=i(A_{t}M_{0}+iB_{t})(C_{t}M_{0}+iD_{t})^{-1}; $$

One proves (see for instance Nazaikiinskii et al. [27]) that

- The phase $\mathcal{\gamma}(t)$, is the symmetrized action integral
  18 $$\mathcal{\gamma}(t)=\int_{0}^{t} \biggl(\frac{1}{2}\sigma(z_{\tau},\dot {z}_{\tau})-H \biggr) d\tau. $$

## Quantum Blobs

Quantum blobs are minimum uncertainty units which are measured using not volume, but rather symplectic capacity, which has the properties of an area—that is of action! Besides the fact that they allow a geometric description of the uncertainty principle [7–11] (of which the reader will find a precise description in next subsection), we are going to see that they are intimately related to the notion of squeezed coherent states, of which it can be considered as a phase space geometric picture.

By definition, a quantum blob is a subset $\mathcal{QB}^{2n}=\mathcal{QB}^{2n}(z_{0},S)$ of $\mathbb{R}_{z}^{2n}$ which can be deformed into the phase space ball $B^{2n}(\sqrt{\hbar}):|z|\leq\hbar$ using only translations and linear canonical transformations $S\in\operatorname{Sp}(2n,\mathbb{R})$. Equivalently, $\mathcal{QB}^{2n}$ is an ellipsoid obtained from $B^{2n}(\sqrt{\hbar})$ by an affine symplectic transformation. More precisely:

### Definition 3

Let $S\in\operatorname{Sp}(2n,\mathbb{R})$ and $z_{0}\in\mathbb{R}_{z}^{2n}$. Then $\mathcal{QB}^{2n}(z_{0},S)=T(z_{0})SB^{2n}(\sqrt{\hbar})$ where *T*(*z*_0_) is the translation operator *z*↦*z*+*z*_0_. Equivalently, it is the set

$$\mathcal{QB}^{2n}(z_{0},S)=\bigl\{z:\bigl(S^{-1}\bigr)^{T}S^{-1}(z-z_{0})^{2}\leq\hbar \bigr\}$$

where we are writing (*S* ^−1^)^*T*^ *S* ^−1^(*z*−*z*_0_)^2^ for (*S* ^−1^)^*T*^ *S* ^−1^(*z*−*z*_0_)^2^. The set of all quantum blobs in phase space $\mathbb{R}_{z}^{2n}$ is denoted $\mathcal{QB}(2n,\mathbb{R})$.

One shows (de Gosson [11], de Gosson and Luef [15]) that a quantum blob $\mathcal{QB}^{2n}(z_{0},S)$ is characterized by the two following *equivalent* properties:

- *The intersection of the ellipsoid* $\mathcal{QB}^{2n}(z_{0},S)$ *with a plane passing through* *z*_0_ *and parallel to any of the plane of canonically conjugate coordinates* *x*_*j*_,*p*_*j*_ *in* $\mathbb{R}_{z}^{2n}$ *is an ellipse with area* $\pi(\sqrt{\hbar})^{2}=\frac{1}{2}h$; *that area is called the* symplectic capacity *of the quantum blob* $\mathcal{QB}^{2n}(z_{0},S)$ (*we will discuss more in detail this notion in a moment*);
- *The supremum of the set of all numbers* *πR* ^2^ *such that the ball* $B^{2n}(\sqrt{R}):|z|\leq R$ *can be embedded into* $\mathcal{QB}^{2n}(z_{0},S)$ *using canonical transformations* (*linear, or not*) *is* $\pi(\sqrt{\hbar})^{2}$. *Hence no phase space ball with radius* $R>\sqrt{\hbar}$ *can be* “*squeezed*” *inside* $\mathcal{QB}^{2n}(z_{0},S)$ *using only canonical transformations* (G*romov’s non-squeezing theorem* [20], *alias the principle of the symplectic camel*).

It turns out (de Gosson [11]) that in the first of these conditions one can replace the plane of conjugate coordinates with any symplectic plane (a symplectic plane is a two-dimensional subspace of $\mathbb{R}_{z}^{2n}$ on which the restriction of the symplectic form *σ* is again a symplectic form).

Clearly there is a natural action of symplectic matrices on quantum blobs: for $S^{\prime}\in\operatorname{Sp}(2n,\mathbb{R})$ we have *S*′*T*(*z*_0_)=*T*(*S* ^′−1^ *z*_0_)*S*′ and hence

19 $$S^{\prime}\bigl[\mathcal{QB}^{2n}(z_{0},S)\bigr]=T\bigl(S^{\prime-1}z_{0}\bigr)S^{\prime}SB^{2n}(\sqrt{\hbar})=\mathcal{QB}^{2n}\bigl(z_{0},S^{\prime}S\bigr). $$

Conversely:

### Proposition 4

*Let* $G\in\operatorname{Sp}(2n,\mathbb{R})$ *be positive*-*definite and symmetric*. *The set* {*z*:*G*(*z*−*z*_0_)^2^≤*ħ*} *is a quantum blob* $\mathcal{QB}^{2n}(z_{0},S)$.

### Proof

As a consequence of the symplectic polar decomposition theorem (see e.g. de Gosson [11]) there exists $S\in\operatorname{Sp}(2n,\mathbb{R})$ such that *G*=(*S* ^−1^)^*T*^ *S* ^−1^ hence the condition *G*(*z*−*z*_0_)^2^≤*ħ* is equivalent to (*S* ^−1^)^*T*^ *S* ^−1^(*z*−*z*_0_)^2^≤*ħ*. □

The symplectic matrix *S* defining a given quantum blob is not unique; one shows (see de Gosson [11]) that $\mathcal{QB}^{2n}(z_{0},S)=\mathcal{QB}^{2n}(z_{0},S^{\prime})$ if and only if *S*′=*SU* where *U* is a symplectic rotation, i.e. an element of the subgroup $U(n)=\operatorname{Sp}(2n,\mathbb{R})\cap O(2n,\mathbb{R})$ of the symplectic group. This property reflects the invariance of phase space balls centered at the origin under rotations. A consequence of this fact is that we have the following topological identification (de Gosson [10]):

$$\mathcal{QB}(2n,\mathbb{R})\equiv\mathbb{R}^{n(n+1)}\times \mathbb{R}^{2n}\equiv\mathbb{R}^{n(n+3)}.$$

Thus, if we view $\mathcal{QB}(2n,\mathbb{R})$ as a “quantum phase space” its topological dimension *n*(*n*+3) is much larger than that, 2*n*, of the classical phase space, even when *n*=1 (in the latter case $\dim\mathcal{QB}(2,\mathbb{R})=3$, which is easily understood as follows: one need one parameter to specify the centre of the quantum blob (which is here an ellipse with area *h*/2), one to specify one of the principal axes, and another to describe the angle of a principal axe with, say, the *x*-axis. A similar interpretation applies in higher dimensions.

Let us briefly compare quantum blobs to the usual quantum cells from statistical mechanics. A quantum cell is typically a phase space cube with volume $(\sqrt{h})^{2n}=h$. The first obvious remark is that these cells do not have any symmetry under general symplectic transformations; while such a transformation preserves volume, a cube will in general be distorted into a multidimensional polyhedron. But what is more striking is the comparison of volumes. Since a quantum blob is obtained from the ball $B^{2n}(\sqrt{\hbar})$ by a volume-preserving transformation its volume is given by

$$\operatorname{Vol} \bigl( \mathcal{QB}^{2n}(z_{0},S) \bigr)=\frac{h^{n}}{n!2^{n}}$$

and is hence *n*!2^*n*^ smaller than that of a quantum cell. For instance, in the case of the physical three-dimensional configuration space this leads to a factor of 48. In the case of a macroscopic system with *n*=10^23^ this fact becomes unimaginably large. This is in strong contrast with the fact that the orthogonal projection of a quantum blob on any plane *x*_*j*_,*p*_*j*_ of conjugate coordinates (or, more generally, on any symplectic plane) is an ellipse with area equal to *πħ*=*h*/2.

## The Correspondence $\mathcal{G}$

Recall that the Wigner transform of a pure state Ψ is given by

20 $$W\Psi(z)= \biggl( \frac{1}{2\pi\hbar} \biggr)^{n}\int e^{-\frac{i}{\hbar }p\cdot y}\Psi\biggl(x+\frac{1}{2}y\biggr)\Psi^{\ast}\biggl(x-\frac{1}{2}y\biggr)d^{n}y, $$

where the star ^∗^ denotes complex conjugation.

The Wigner transform of the fiducial coherent state Φ^*ħ*^ is given by

21 $$W\Phi^{\hbar}(z)=(\pi\hbar)^{-n}e^{-\frac{1}{\hbar}|z|^{2}}. $$

More generally [11, 26] the Wigner transform

22 $$W\Phi_{M}^{\hbar}(z)= \biggl( \frac{1}{2\pi\hbar}\biggr)^{n}\int e^{-\frac{i}{\hbar}p\cdot y}\Phi_{M}^{\hbar}\biggl(x+\frac{1}{2}y\biggr)\Phi_{M}^{\hbar }\biggl(x-\frac{1}{2}y\biggr)^{\ast}d^{n}y $$

of the squeezed coherent state $\Phi_{M}^{\hbar}=\Phi_{X,Y}^{\hbar}$ is given by the formula:

23 $$W\Phi_{M}^{\hbar}(z)=(\pi\hbar)^{-n}e^{-\frac{1}{\hbar}Gz\cdot z}, $$

where *G* is the real 2*n*×2*n* matrix 24 Notice that *G* does not contain the parameter *ħ*. It turns out that *G* is both positive definite and symplectic; in fact *G*=*S* ^*T*^ *S* where 25 The same analysis applies to $\Phi_{M,z_{0}}^{\hbar}(z_{0})$. Letting the translation operator *T*(*z*_0_):*z*↦*z*+*z*_0_ act on functions on phase space by the rule *T*(*z*_0_)*f*(*z*)=*f*(*z*−*z*_0_) and its quantum variant, the Heisenberg–Weyl operator (26) we have the translational property

26 $$W\bigl(\widehat{T}^{\hbar}(z_{0})\psi\bigr)(z)=T(z_{0})W(\psi) (z) $$

and hence, in particular

27 $$W\Phi_{M,z_{0}}^{\hbar}(z)=(\pi\hbar)^{-n}e^{-\frac{1}{\hbar}G(z-z_{0})^{2}}. $$

Let us now state and prove the following essential correspondence result which *identifies* squeezed coherent states with quantum blobs:

### Proposition 5

*There is a bijective correspondence*

$$\mathcal{G}:\mathcal{CS(}n,\mathbb{R})\longleftrightarrow\mathcal{QB}(2n,\mathbb{R})$$

*between squeezed coherent states and quantum blobs*. *That correspondence is defined as follows*: *if*

$$W\Phi_{M,z_{0}}^{\hbar}(z)=(\pi\hbar)^{-n}e^{-\frac{1}{\hbar}G(z-z_{0})^{2}}$$

*then we have*

28 $$\mathcal{G}\bigl[\Phi_{M,z_{0}}^{\hbar}\bigr]=\bigl \{z:G(z-z_{0})^{2}\leq\hbar\bigr\}=\mathcal{QB}^{2n}\bigl(z_{0},S^{-1}\bigr), $$

*where the symplectic matrix* *S* *is given by formula* (25) *above*.

### Proof

While the definition of the correspondence $\mathcal{G}$ is straightforward, it is not immediately clear why it should be bijective. Let us first show that it is one-to-one. Suppose that $\mathcal{G}[\Phi_{M,z_{0}}^{\hbar}]=\mathcal{G}[\Phi_{M^{\prime},z_{0}^{\prime}}^{\hbar}]$, that is

$$\bigl\{z:G(z-z_{0})^{2}\leq\hbar\bigr\}=\bigl \{z:G^{\prime}\bigl(z-z_{0}^{\prime}\bigr)^{2}\leq \hbar\bigr\}.$$

We must then have *G*=*G*′ and $z_{0}=z_{0}^{\prime}$ so that $W\Phi_{M,z_{0}}^{\hbar}(z)=W\Phi_{M^{\prime},z_{0}^{\prime}}^{\hbar}(z)$; since the Wigner transform of a function Ψ determines uniquely determines Ψ up to a unimodular factor we have $\Phi_{M^{\prime},z_{0}^{\prime}}^{\hbar}=e^{\frac{i}{\hbar}\gamma}\Phi_{M,z_{0}}^{\hbar}$ for some real phase *γ*. Let us next show that $\mathcal{G}$ is onto; this will at the same time yield a procedure for calculating the inverse of $\mathcal{G}$. Assume that $\mathcal{QB}^{2n}(0,S^{-1})=S^{-1}B^{2n}(\sqrt{\hbar})$ is a quantum blob centered at the origin. One can factorize the matrix *S* ^−1^ as follows (“pre-Iwasawa factorization”; cf. [11], Sect. 2.2, Corollary 2.30): where the symmetric matrix *L* is given by

29 $$L=\bigl(D^{T}D+B^{T}B\bigr)^{1/2} $$

is symmetric positive definite,

30 $$Q=-\bigl(C^{T}D+A^{T}B\bigr) \bigl(D^{T}D+B^{T}B\bigr)^{-1/2} $$

with *A*+*iB*∈*U*(*n*,ℂ). The matrix  is thus a symplectic rotation and, as such, leaves any ball centered at the origin invariant. Setting *X* ^1/2^=*L* and *Y*=*X* ^1/2^ *Q* it follows that we have  the quantum blob $\mathcal{QB}^{2n}(0,S^{-1})$ is thus represented by *Gz*⋅*z*≤*ħ* where *G*=*S* ^*T*^ *S* is of the type (24); define now $\Phi_{M}^{\hbar}=\Phi_{X,Y}^{\hbar}$ by assigning to *X* and *Y* the values *L* ^2^ and *X* ^1/2^ *Q* found above. The argument generalizes in a straightforward way to quantum blobs with arbitrary centre. □

In view of the correspondence between squeezed coherent states and quantum blobs, we can give a phase space picture of formula (16) for the time evolution of a squeezed coherent state when the Hamiltonian function is quadratic. Let us study this deformation in some detail.

We claim that an initial quantum blob becomes after time *t* a new quantum blob which is just its image by the classical flow *S*_*t*_:

### Proposition 6

*After time* *t* *the initial quantum blob* $\mathcal{QB}^{2n}(z_{0},S_{0})$ *becomes the quantum blob*

$$S_{t}\bigl[\mathcal{QB}^{2n}(z_{0},S_{0})\bigr]=\mathcal{QB}^{2n}(z_{t},S_{0}S_{t}).$$

*Thus*, *the quantum motion of squeezed coherent states induces the classical motion for the corresponding quantum blob*.

### Proof

At initial time we are in presence of an initial quantum blob $\mathcal{QB}^{2n}(z_{0},S)$, set of all phase space points *z* such that *G*(*z*−*z*_0_)(*z*−*z*_0_)≤*ħ* with *G*=(*S* ^−1^)^*T*^ *S* ^−1^. Let us calculate the Wigner transform

31 $$W\Psi(z,t)= \biggl( \frac{1}{2\pi\hbar} \biggr)^{n}\int e^{-\frac{i}{\hbar }p\cdot y}\Psi\biggl(x+\frac{1}{2}y,t\biggr)\Psi^{\ast}\biggl(x-\frac{1}{2}y,t\biggr)d^{n}y $$

of the solution Ψ(*z*,*t*) of Schrödinger’s equation (13). Using formula (15)) together with the symplectic covariance of the Wigner transform (de Gosson [11]) we have

$$W\Psi(z,t)=W\bigl(\widehat{S}_{t}\Phi_{M_{0},z_{0}}^{\hbar}\bigr) (z)=W\Phi_{M_{0},z_{0}}^{\hbar}\bigl(S_{t}^{-1}z\bigr)$$

that is, in view of formula (23) giving the Wigner transform of $\Phi_{M_{0},z_{0}}^{\hbar}$: 32
33 It follows that the initial quantum blob has become the ellipsoid defined by

$$\bigl(S_{t}^{-1}\bigr)^{T}G_{0}S_{t}^{-1}(z-z_{t})^{2}\leq\hbar $$

which proves our claim. □

## Statistical Interpretation of $\mathcal{G}$

We begin by recalling the notion of symplectic capacity, which was already mentioned briefly in the beginning of this paper after the definition of quantum blobs. See Hofer–Zehnder [25], Polterovich [29], or de Gosson [12] and de Gosson and Luef [15] for a review of this notion from point of view easily accessible to physicists.

A symplectic capacity on phase space $\mathbb{R}_{z}^{2n}$ assigns to every subset Ω of $\mathbb{R}_{z}^{2n}$ a number *c*(Ω)≥0, or +∞. This assignment must obey the following rules:

1. If Ω⊂Ω′ then *c*(Ω)≤*c*(Ω′);
2. If *f* is a canonical transformation then *c*(*f*(Ω))=*c*(Ω);
3. If *λ* is a real number then *c*(*λ*Ω)=*λ* ^2^ *c*(Ω); here *λ*Ω is the set of all points *λz* when *z*∈Ω;
4. We have $c(B^{2n}(R))=\pi R^{2}=c(Z_{j}^{2n}(R))$; here *B* ^2*n*^(*R*) is the ball |*x*|^2^+|*p*|^2^≤*R* ^2^ and $Z_{j}^{2n}(R)$ the cylinder $x_{j}^{2}+p_{j}^{2}\leq R^{2}$.

There exist infinitely many symplectic capacities, however the construction of any of them is notoriously difficult (the fact that symplectic capacities exist is actually equivalent to Gromov’s non-squeezing theorem [20]). However they all agree on phase space ellipsoids. In fact:

### Proposition 7

*Let* $\mathcal{W}:Mz\cdot z\leq\hbar$ *where* *M* *is a symmetric positive definite* 2*n*×2*n* *matrix*. *We have*

34 $$c(\mathcal{W})=\pi\hbar/\lambda_{\max} $$

*for every symplectic capacity c*; *here* *λ*_max_ *is the largest symplectic eigenvalue of M*.

The proof of this result is based on a symplectic diagonalisation of *M*; see de Gosson [11], Hofer–Zehnder [25], Polterovich [29], and the references therein. Recall that the symplectic eigenvalues of *M* are defined as follows: the eigenvalues of the matrix *JM* are of the type ±*iλ*_*j*_ with *λ*_*j*_>0; the sequence (*λ*_1_,…,*λ*_*n*_) is then the symplectic spectrum of *M* and the *λ*_*j*_ the symplectic eigenvalues.

The smallest symplectic capacity is denoted by *c*_min_ (“Gromov width”): by definition *c*_min_(Ω) is the supremum of all numbers *πR* ^2^ such that there exists a canonical transformation such that *f*(*B* ^2*n*^(*R*))⊂Ω. The fact that *c*_min_ really is a symplectic capacity follows from Gromov’s [20] symplectic non-squeezing theorem. For a discussion of Gromov’s theorem (and comments) from the physicist’s point of view see de Gosson [12], de Gosson and Luef [15].

Let now *K* be an arbitrary real symmetric positive-definite matrix of order 2*n* and define the normalized phase space Gaussian

$$W_{K}(z)=(\pi\hbar)^{-n/2}(\det K)^{1/2}e^{-\frac{1}{\hbar}Kz\cdot z}.$$

When $K=G\in\operatorname{Sp}(2n,\mathbb{R})$ the Gaussian *W*_*K*_(*z*) is the Wigner transform of some squeezed coherent state. Following Littlejohn [26] we define a matrix Σ by the relation

35 $$\Sigma=\frac{\hbar}{2}K^{-1} $$

hence *W*_*K*_(*z*) takes the familiar form

$$W_{K}(z)=(2\pi)^{-n}(\det\Sigma)^{-1/2}e^{-\frac{1}{2}\Sigma^{-1}z\cdot z}$$

suggesting to interpret Σ as the covariance matrix of a normal probability distribution centered at the origin. We will write Σ in block form where each block has dimension *n*×*n* and Δ(*P*,*X*)=Δ(*X*,*P*)^*T*^; we use the notation  and set

$$(\Delta x_{j})^{2}=\operatorname{Cov}(x_{j},x_{j}), \quad (\Delta p_{j})^{2}=\operatorname{Cov}(p_{j},p_{j})$$

for 1≤*j*≤*n*. The essential observation we make is:

### Proposition 8

*Consider the phase space ellipsoid* $\mathcal{W}:\frac{1}{2}\Sigma^{-1}z\cdot z\leq1$. *The topological condition*

36 $$c(\mathcal{W)\geq}\frac{1}{2}\hbar $$

*implies the Robertson–Schrödinger inequalities*

37 $$(\Delta x_{j})^{2}(\Delta p_{j})^{2}\geq\operatorname{Cov}(x_{j},p_{j})^{2}+\frac{1}{4}\hbar^{2} $$

*for* 1≤*j*≤*n* *hence*, *in particular*, *the Heisenberg uncertainty relations* $\Delta x_{j}\Delta p_{j}\geq\frac{1}{2}\hbar$.

The proof of this important result is given in de Gosson [10–12] (also see de Gosson and Luef [15]). It is based on the following fact, well-known in the quantum optics community: the condition

38 $$\Sigma+\frac{i\hbar}{2}J\text{ is Hermitian positive semi-definite} $$

implies the Robertson–Schrödinger inequalities (37) (but it is not equivalent to it: see de Gosson [12] for a counterexample). Some algebra together with a formula giving the symplectic capacity of an ellipsoid, then shows that conditions (38) and (36) are equivalent. Notice that the matrix $\Sigma+\frac{i\hbar}{2}J$ is always Hermitian since $(\Sigma+\frac{i\hbar}{2}J)^{\ast}=\Sigma-\frac{i\hbar}{2}J^{T}$ and *J* ^*T*^=−*J*. We mention that symplectic capacities can be used as well for the study of the more general uncertainty principle related to non-commutative quantum mechanics as we have shown in de Gosson [13].

Suppose now that the covariances defined above correspond to some quantum state Ψ (pure or mixed). The Robertson–Schrödinger inequalities (37) are saturated (i.e. they become equalities) exactly when that state is a squeezed coherent state $\Phi_{M}^{\hbar}$ where *M*=*X*+*iY* is determined via the Wigner transform of $\Phi_{M}^{\hbar}$ (cf. (35))

$$W\Phi_{M}^{\hbar}(z)=(\pi\hbar)^{-n}e^{-\frac{1}{\hbar}Gz\cdot z},\quad G=\frac{\hbar}{2}\Sigma^{-1}.$$

For instance if $\Phi_{M}^{\hbar}$ is the fiducial coherent state Φ^*ħ*^ all the covariances vanish and the inequalities (37) reduce to $\Delta x_{j}\Delta p_{j}=\frac{1}{2}\hbar$ for 1≤*j*≤*n*.

## Fermi’s Function *g*_F_

In a largely forgotten paper from 1930 Fermi [17] associates to every quantum state Ψ a certain hypersurface *g*_F_(*x*,*p*)=0. Fermi’s paper has recently been rediscovered by Benenti and Strini [3] and Benenti [2]; in particular these authors give a heuristic comparison of the function *g*_F_ and the Wigner transform *W*Ψ. Let us shortly study the relationship between Fermi’s function and the notion of quantum blob. The starting point is Fermi’s observation that the state of a quantum system may be defined in two different (but equivalent) ways, namely by its wavefunction Ψ or by measuring a certain physical quantity whose definition goes as follows. Writing the wavefunction in polar form Ψ(*x*)=*R*(*x*)*e* ^*i*Φ(*x*)/*ℏ*^ (*R*(*x*)≥0 and Φ(*x*) real) one verifies by a straightforward calculation that Ψ is a solution of the partial differential equation

39 $$\widehat{g_{\mathrm{F}}}\Psi=0, $$

where

40 $$\widehat{g_{\mathrm{F}}}= ( -i\hbar\nabla_{x}-\nabla_{x}\Phi )^{2}+\hbar^{2}\frac{\nabla_{x}^{2}R}{R}. $$

Equation (39) seems at first sight to be ad hoc and somewhat mysterious. However much of the mystery disappears if one remarks that it is obtained by the gauge transform *p*⟶*p*−∇_*x*_Φ from the trivial equation

41 $$\biggl( -\hbar^{2}\nabla_{x}^{2}+\hbar^{2}\frac{\nabla_{x}^{2}R}{R} \biggr) R=0 $$

satisfied by the amplitude *R*.

Consider now the Weyl symbol of the operator $\widehat{g_{\mathrm{F}}}$; it is the real function

42 $$g_{\mathrm{F}}(x,p)= ( p-\nabla_{x}\Phi )^{2}+\hbar^{2}\frac{\nabla_{x}^{2}R}{R}. $$

When $\nabla_{x}^{2}R/R<0$ the equation *g*_F_(*x*,*p*)=0 determines a hypersurface $\mathcal{H}_{\mathrm{F}}$ in phase space $\mathbb{R}_{z}^{2n}$ which Fermi ultimately *identifies* with the state Ψ itself. Let us examine the relation between Fermi’s Ansatz and the notion of quantum blob we have introduced in this paper. Let $\Phi_{M}^{\hbar}=\Phi_{X,Y}^{\hbar}$ be the squeezed coherent state defined by (2); we have in this case $\Phi(x)=-\frac{1}{2}Yx\cdot x$ and *R*(*x*)=*e* ^−*Xx*⋅*x*/2*ħ*^ hence Fermi’s function is given by

43 $$g_{\mathrm{F}}(x,p)= ( p+Yx )^{2}+X^{2}x\cdot x-\hbar \operatorname{Tr}X, $$

where $\operatorname{Tr}X$ is the trace of the matrix *X* (note that $\operatorname{Tr}X>0$ since *X* is positive definite). The corresponding hypersurface $\mathcal{H}_{\mathrm{F}}$ is thus the phase space set44 Recall now that the Wigner transform of $\Phi_{M}^{\hbar}$ is the function $W\Phi_{M}^{\hbar}(z)= (\pi\hbar)^{-n}e^{-\frac{1}{\hbar}Gz\cdot z}$ where (formulas (24) and (25)) 45 and *S* is the symplectic matrix 46 An immediate calculation shows that the matrices *M*_F_ and *G* are related by the formula47


Let us consider the “Fermi ellipsoid” $\mathcal{W}_{\mathrm{F}}:M_{\mathrm{F}}z\cdot z\leq\hbar$ bounded by the hypersurface $\mathcal{H}_{\mathrm{F}}$.

### Proposition 9

(*i*) *There exist symplectic coordinates in which the Fermi ellipsoid* $\mathcal{W}_{\mathrm{F}}:M_{\mathrm{F}}z\cdot z\leq\hbar$ *is represented by the inequality*

48 $$Xx\cdot x+Xp\cdot p\leq\hbar\operatorname{Tr}X $$

*or by the inequality*

49 $$\sum_{j=1}^{N}\lambda_{j}\bigl(x_{j}^{2}+p_{j}^{2}\bigr)\leq \hbar\operatorname{Tr}X, $$

*where* *λ*_1_,…,*λ*_*n*_ *are the eigenvalues of* *X*;

(*ii*) *We have*

50 $$c(\mathcal{W}_{\mathrm{F}})=\frac{\pi\operatorname{Tr}X}{\lambda_{\max}}\hbar\geq \frac{1}{2}h $$

*where* *λ*_max_ *is the largest eigenvalue of* *M*_F_ *and*

51 $$\frac{1}{2}h\leq c(\mathcal{W}_{\mathrm{F}})\leq\frac{nh}{2}. $$

### Proof

(i) In view of (47) the inequality *M*_F_ *z*⋅*z*≤*ħ* is equivalent to  with *u*=*Sz*. Let *U* be a rotation in ℝ^*n*^ diagonalising *X*, that is *X*=*U* ^*T*^ *DU* with $D=\operatorname{diag}(\lambda_{1}, \ldots, \lambda_{n})$. Setting  the inequality *M*_F_ *z*⋅*z*≤*ħ* is now equivalent to (49) and one concludes by noting that the matrix  is in *U*(*n*) (i.e. *R* is a symplectic rotation). (ii) Since symplectic capacities are invariant by symplectic transformations, it suffices to prove formula (50) when $\mathcal{W}_{\mathrm{F}}$ is given by (48) or by (49). In view of Proposition 7 we have $c(\mathcal{W}_{\mathrm{F}}\mathcal{)=}\pi\hbar/\lambda_{\max}$ and the equality in (50) follows noting that the symplectic spectrum of *X* consists of precisely the eigenvalues of *X*. The double inequality (51) is obvious since $\lambda_{\max}\leq\operatorname{Tr}X\leq nh/2$. □

In view of the double inequality (51) Fermi ellipsoids are not in general quantum blobs (except for *n*=1). However each of these ellipsoids contains a quantum blob centered at the origin. To see this it suffices to show that the ellipsoid defined by (49) contains the ball $B^{2n}(\sqrt{\hbar})$ (because the image of a quantum blob by a linear symplectic transformation is again a quantum blob). Now, if (*x*,*p*) is in $B^{2n}(\sqrt{\hbar})$ then

$$\sum_{j=1}^{N}\frac{\lambda_{j}}{\operatorname{Tr}X}\bigl(x_{j}^{2}+p_{j}^{2}\bigr)\leq\sum_{j=1}^{N}\bigl(x_{j}^{2}+p_{j}^{2}\bigr)\leq \hbar $$

since $\lambda_{j}/\operatorname{Tr}X\leq1$ hence our claim.

Let us discuss the results above on a few simple examples. For the fiducial coherent state $\Phi^{\hbar}(x)=(\pi\hbar)^{-n/4}e^{-|x|^{2}/2\hbar}$ we have *X*=*I* and *Y*=0 hence the Fermi ellipsoid $\mathcal{W}_{\mathrm{F}}$ is the disk |*x*|^2^+|*p*|^2^≤*nħ* whose symplectic capacity is *nπħ*=*nh*/2. The operator (40) is here

52 $$\widehat{g_{\mathrm{F}}}=-\hbar^{2}\nabla_{x}^{2}+|x|^{2}-n\hbar $$

and the relation $\widehat{g_{\mathrm{F}}}\Phi^{\hbar}=0$ is hence equivalent to

53 $$\frac{1}{2}\bigl(-\hbar^{2}\nabla_{x}^{2}+|x|^{2}\bigr)\Phi^{\hbar}=\frac{1}{2}n\hbar\Phi^{\hbar} $$

which simply states the well-known fact that Φ^*ħ*^ is an eigenvector of the harmonic oscillator Hamiltonian $\widehat{H}=\frac{1}{2}(-\hbar^{2}\nabla_{x}^{2}+|x|^{2})$ corresponding to the first energy level $E_{0}=\frac{1}{2}n\hbar$. One easily verifies that if Ψ^*ħ*^ is the tensor product of *n* copies of the (unnormalised) Hermite functions $xe^{-x^{2}/2\hbar}$ then the equation $\widehat{g_{\mathrm{F}}}\Psi^{\hbar }=0$ is equivalent to

54 $$\frac{1}{2}\bigl(-\hbar^{2}\nabla_{x}^{2}+|x|^{2}\bigr)\Psi^{\hbar}=\frac{3}{2}n\hbar\Psi^{\hbar}. $$

The argument may be repeated, and one finds that the Fermi equation (39) corresponding to a Hermite function, is always equivalent to the eigenstate equation for the harmonic oscillator corresponding to that function.

The discussion above can be generalised, using metaplectic covariance properties, to the case of quantum states of operators corresponding to arbitrary Hamiltonians $H=\frac{1}{2}Mz\cdot z$ where *M* is symmetric positive definite (generalised harmonic oscillator). It is certainly worthwhile studying what happens in more general cases where the quantum states are no longer Gaussians; a first step in this direction has been taken by Benenti [2].

## Concluding Remarks and Perspectives

Using the correspondence $\mathcal{G}$ defined in Sect. 4 we have sees that quantum blobs exactly correspond to those quantum states which have minimum uncertainty in the sense of Robertson–Schrödinger. This justifies our claim that quantum blobs represent the smallest regions of phase space which make sense from a quantum-mechanical perspective. In fact, contrarily to what is often believed the Heisenberg inequalities and their stronger version, the Robertson–Schrödinger inequalities (37), are not a statement about the accuracy of our measurement instruments; their derivation assumes on the contrary *perfect instruments*. The correct interpretation of these inequalities is the following (see e.g. Peres [28], p. 93): if the same preparation procedure is repeated a large number of times, and is followed by either by a measurement of *x*_*j*_, or by a measurement of *p*_*j*_, the results obtained have standard deviations Δ*x*_*j*_ and Δ*p*_*j*_ satisfying these inequalities. Such a process thus makes clear the impossibility of talking about points in phase space having some intrinsic meaning (cf. Butterfield’s paper [5] refuting “pointillisme”). We note that in [16] Dragoman uses the partition of phase space in quantum blobs to propose a new formulation of quantum mechanics, based on the following postulates:

### Axiom 10

*It is not possible to localize a quantum particle in a phase space regions smaller that a quantum blob*.

### Axiom 11

*The phase space extent of a quantum particle is smaller than a quantum blob*.

These postulates and their implications for quantum physics certainly deserve to be discussed further.

In a recent paper [14] Hiley and I study a version of the quantum Zeno paradox for the Bohm trajectory of a sharply located particle modelled by a Dirac distribution. We showed in this paper that such a recorded quantum trajectory (in, for instance, a bubble chamber) is just the classical trajectory predicted by standard Hamiltonian mechanics. It would be both very interesting and realistic to study this kind of quantum Zeno effect by replacing the point-like particle by a squeezed coherent state, that is, equivalently, by a quantum blob. A good starting point could be Hiley [21] where the relationship between the Wigner–Moyal and Bohm approaches is elucidated; also the connections with the ideas of Hiley and collaborators in [22–24] could be useful here. We have seen in Proposition 6 that a quantum blob evolves *classically* under the action of the linear Hamiltonian flow determined by a quadratic Hamiltonian. Of course quadratic Hamiltonians are of a very particular type; the result above remains approximately valid for arbitrary physical Hamiltonians, and this with an excellent approximation during generically very large times (Ehrenfest time, as it is called in the theory of quantum revivals). This observation could allow us to prove, using the correspondence $\mathcal{G}$, the following conjecture considerably extending the results in de Gosson and Hiley [14]:

### Conjecture 12

*When we continuously observe the motion of a quantum blob we see its classical Hamiltonian motion*; *i*.*e*. *an initial quantum blob* $\mathcal{QB}^{2n}$ *will be transformed in the set* $f_{t}^{H}(\mathcal{QB}^{2n})$ *after time* *t*; *here* $f_{t}^{H}$ *is the classical Hamilton flow* (*Arnol’d *[1], *Goldstein *[19]).

In Sect. 6 we briefly discussed some elementary properties of the Fermi function $g_{\mathrm{F}}^{\Psi}$ and of the associated Fermi ellipsoid $\mathcal{W}_{\mathrm{F}}$. The discussion was actually limited to Gaussian states. We make the following conjecture:

### Conjecture 13

*Let* Ψ *be a quantum state for which the Fermi equation* *g*_F_(*x*,*p*) *defines a hypersurface in phase space bounding a compact set* Ω_F_. *Then there exists a symplectic capacity* *c* *such that* $c(\Omega_{\mathrm{F}})\geq\frac{1}{2}h$ *and* Ω_F_ *contains a quantum blob*.

The observant Reader will perhaps have noticed that the equation *g*_F_(*x*,*p*)=0 for a system of particles with mass *m* can be rewritten

$$\frac{1}{2m} ( p-\nabla_{x}\Phi )^{2}+Q=0$$

if one introduces the quantum potential

$$Q=-\frac{\hbar^{2}}{2m}\frac{\nabla_{x}^{2}R}{R}$$

familiar from the Bohmian approach top quantum mechanics (see Bohm and Hiley [4]). There thus seems to be a deep connection between this theory and the phase space approach which certainly deserves to be elucidated and extended.

I am sure that Basil will be excited by these possibilities, and I look forward writing new papers with him about the truly fascinating topic of quantum phase space!

Happy birthday, Basil!
